# Whole Exome Sequencing of Rapid Autopsy Tumors and Xenograft Models Reveals Possible Driver Mutations Underlying Tumor Progression

**DOI:** 10.1371/journal.pone.0142631

**Published:** 2015-11-10

**Authors:** Tao Xie, Monica Musteanu, Pedro P. Lopez-Casas, David J. Shields, Peter Olson, Paul A. Rejto, Manuel Hidalgo

**Affiliations:** 1 Oncology Research, Pfizer Worldwide Research and Development, San Diego, California, United States of America; 2 CNIO (Spanish National Cancer Research Centre), E-28029, Madrid, Madrid, Spain; Garvan Institute of Medical Research, AUSTRALIA

## Abstract

Pancreatic Ductal Adenocarcinoma (PDAC) is a highly lethal malignancy due to its propensity to invade and rapidly metastasize and remains very difficult to manage clinically. One major hindrance towards a better understanding of PDAC is the lack of molecular data sets and models representative of end stage disease. Moreover, it remains unclear how molecularly similar patient-derived xenograft (PDX) models are to the primary tumor from which they were derived. To identify potential molecular drivers in metastatic pancreatic cancer progression, we obtained matched primary tumor, metastases and normal (peripheral blood) samples under a rapid autopsy program and performed whole exome sequencing (WES) on tumor as well as normal samples. PDX models were also generated, sequenced and compared to tumors. Across the matched data sets generated for three patients, there were on average approximately 160 single-nucleotide mutations in each sample. The majority of mutations in each patient were shared among the primary and metastatic samples and, importantly, were largely retained in the xenograft models. Based on the mutation prevalence in the primary and metastatic sites, we proposed possible clonal evolution patterns marked by functional mutations affecting cancer genes such as KRAS, TP53 and SMAD4 that may play an important role in tumor initiation, progression and metastasis. These results add to our understanding of pancreatic tumor biology, and demonstrate that PDX models derived from advanced or end-stage likely closely approximate the genetics of the disease in the clinic and thus represent a biologically and clinically relevant pre-clinical platform that may enable the development of effective targeted therapies for PDAC.

## Introduction

Pancreatic Ductal Adenocarcinoma (PDAC) is a highly lethal malignancy that represents a major therapeutic challenge[1]. In the United States, PDAC ranks fourth in mortality with a median survival of only six to nine months. The overall five-year survival rate is less than 5%, and even for patients who have surgical resection, the rate is still less than ~25% [2]. The poor prognosis for patients with PDAC partially stems from its propensity to rapidly metastasize [3]. Despite decades of basic and translational research, very few new therapies have been approved. To help develop novel targeted therapeutic strategies to treat PDAC patients, it is critical to understand the cellular and molecular mechanisms that facilitate metastasis formation, and to generate models that more faithfully represent the end-stage disease. Previous studies [4–7] found that PDAC tumors have numerous somatic alterations that affect key oncogenic drivers and tumor suppressor genes including KRAS, TP53, CDKN2A and SMAD4, although it is still not clear which mutations lead to the acquisition of metastatic ability.

Our understanding of the mutational landscape in pancreatic cancer has evolved with larger data sets, newer sequencing technologies and more advanced bioinformatics analyses [8]. In addition to the aforementioned mutated pancreatic cancer genes, lower incidence mutations and structural rearrangements have emerged more recently including mutations in genes involved in DNA repair, axon guidance, receptor tyrosine kinases and epigenetic modifiers [6, 7, 9]. While gemcitabine plus Abraxane and FOLFIRINOX have recently been approved for pancreatic cancer, response rates are still at or below 50% which may be due to additional genetic loci that confer sensitivity or resistance to therapy[10, 11]. Mutational heterogeneity has also been observed in pancreatic cancer. Comparing mutational profiles between primary tumors and paired metastases has been shown to afford an opportunity to identify mutations that may underlie the metastatic process [12–15]. Mutational heterogeneity may also at least partially explain the short-lived responses to virtually all drugs as patients may harbor pre-existing, therapy-resistant clones.

PDX (patient-derived xenograft) models have emerged as preclinical models that better reflect human cancer since the heterogeneity of the tumor more closely reflects patient tumors than traditional cell line xenografts [16]. A key gap in the field has been to generate and characterize models across the spectrum of disease stages [17–19]. Since most clinical trials are tested in late-stage disease, PDX models derived from advanced or end-stage likely represent a closer approximation to the clinic compared to resection-derived models which represent the majority of models generated in the field to date [20, 21]

In this study, we sought to comprehensively characterize somatic SNVs (single-nucleotide variations) in end stage pancreatic cancer and performed whole exome sequencing (WES) on paired primary tumors and metastases. Through this analysis, we identified potential molecular cancer drivers and related dysregulated pathways that might underlie the progression of PDAC. PDX models derived from the same primary and metastatic tumor samples were also sequenced to assess differences in the mutation profiles between primary tumor samples and xenograft models.

## Results

### Reads data generated by whole exome sequencing

Three PDAC patients (patient ID: P042, P047 and P059) were characterized in this study by DNA sequencing of tumors obtained at autopsy, as well as derived xenograft models. We conducted whole-exome sequencing (WES) for 13 samples, including ten tumor samples plus three normal DNA samples from each patient’s peripheral blood (**Table** 1) and generated about 40–50 millions of sequencing read pairs (100bp, paired end) for each sample. After raw reads QC and removal of mouse reads from the PDX samples, we got on average 37.8 million read pairs per sample and mapped the majority (close to 98%) to the human reference genome (human NCBI build 37), which gives an average sequencing mean depth of greater than 120x on covered exonic regions.

**Table 1:** Summary statistics of WES data.

Sample Type	Short Name	%mouse	Read Pairs (in mil.) *	Mean depth
**P042 **				
Normal	P042_NOR	-	43.7	141.0
Primary	P042_PRI	-	57.1	184.4
Primary PDX	P042_PRI_PDX	6.0%	37.3	120.4
Liver metastasis	P042_LIV	-	30.0	96.9
Liver metastasis PDX	P042_LIV_PDX	17.9%	35.0	112.9
**P047**				
Normal	P047_NOR	-	34.6	111.6
Primary	P047_PRI	-	38.0	122.8
Liver metastasis	P047_LIV	-	34.9	112.5
Liver metastasis PDX	P047_LIV_PDX	10.5%	35.7	115.2
Peritoneum metastasis	P047_PER	-	44.7	144.4
**P059**		-		
Normal	P059_NOR	-	32.6	105.4
Primary PDX	P059_PRI_PDX	7.1%	34.9	112.8
Liver metastasis PDX	P059_LIV_PDX	2.4%	32.7	105.6

*For PDX samples, predicted mouse reads were excluded.

### Overview of somatic SNVs

**Fig 1:**
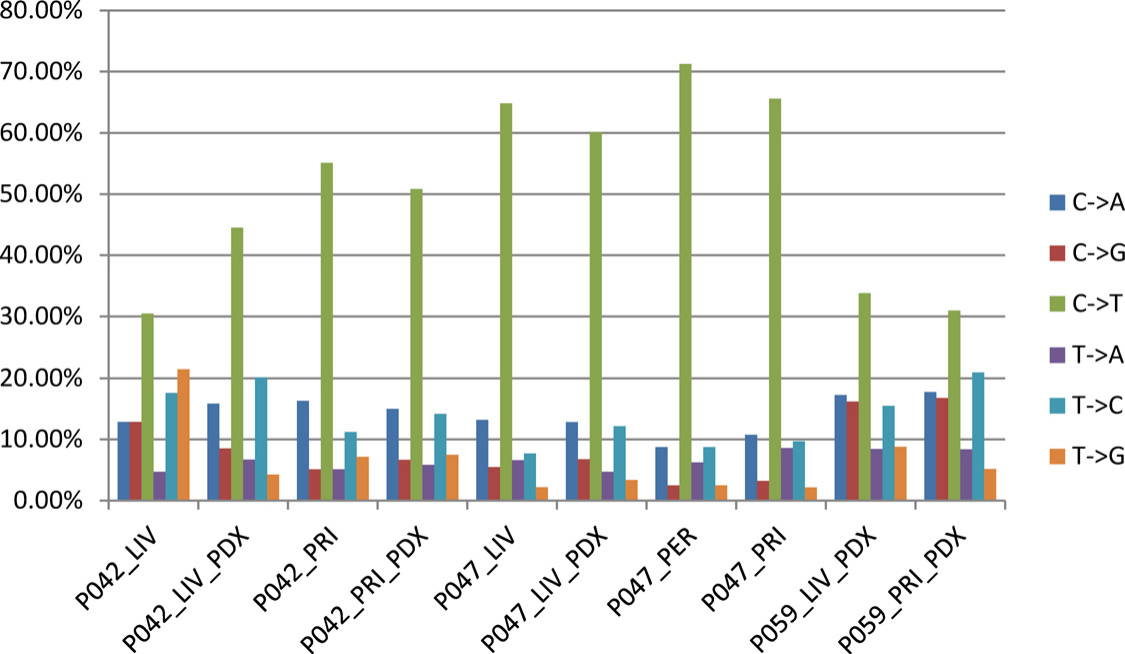
Somatic mutation patterns of primary, metastatic and PDX tumors. Proportion of somatic SNVs by class (C->A, C->G, C->T, T->A, T->C and T-G) in the primary, metastatic and PDX tumor samples are shown for the ten cancer samples included in this study.

By comparing to matched normal DNA, we initially identified an average of 160 somatic SNVs (single nucleotide variants or point mutations) per cancer sample, ranging from as few as 80 (P047_PER) to as many as 312 (P059_PRI_PDX). There were significantly more C>T variations (transitions) in every sample included in this study (Fig 1), similar to what has been reported elsewhere (for example: [22]). The paired primary and metastatic tumors as well as matched tumor and xenograft samples had similar mutation class distributions in most cases: e.g. P047_PRI has a very similar mutation pattern as the two metastatic samples (P047_PER, peritoneum and P047_LIV, liver) from the same patient; for patient P059, though no tumor samples were available for WES analysis, the mutation patterns of the two xenografts (P059_PRI_PDX and P059_LIV_PDX) are more similar to each other than to any sample from different patients. These results suggest that the underlying mutational processes remain the same in primary and metastatic lesions, as well as in their xenograft models. PDX samples tend to have a slightly lower proportion of C>T transitions than their originating tumors. The only exception is P042_LIV which shows substantially more T->G variation (and thus proportionally much less C>T transitions) than any other sample in this study.

We classified mutations into several different categories based on their location (**Table 2**). In general, the majority of somatic mutations are in exonic regions, including UTRs and CDS regions where a subset can alter encoded protein sequences (including missense and non-sense mutations, predicted by the Sift tool [23]), and are likely to have a functional impact. We found on average 55 somatic protein-altering SNVs per sample, ranging from as few as 35 (P047_PER/P047_PRI) to as many as 99 (P059_PRI_PDX). Detailed information can be found in **S1 Table**.

**Table 2:** Summary of locations of somatic SNVs.

Patient		P042				P047			P059	
Category	LIV	LIV_PDX	PRI	PRI_PDX	LIV	LIV_PDX	PER	PRI	LIV_PDX	PRI_PDX
3’_UTR	3	2	5	2	1	0	0	0	7	7
5’_UTR	1	1	1	1	1	1	1	1	3	3
CDS	68	74	44	53	48	62	39	43	102	131
Downstream	12	3	2	3	3	4	3	2	9	8
Intergenic	2	0	0	0	0	1	0	2	6	14
Intronic	97	42	26	32	21	50	20	28	90	98
Splice_site	6	6	6	4	3	6	5	4	9	8
Upstream	11	6	4	6	3	6	1	3	17	12
Other types	33	30	10	19	11	18	11	10	44	31
**Total:**	**233**	**164**	**98**	**120**	**91**	**148**	**80**	**93**	**287**	**312**

Note: UTR, untranslated region; CDS, coding sequence.

### Common and distinct somatic SNVs between primary, metastatic tumors and derived xenografts

Characterization of common and distinct genetic changes between primary and metastatic tumors is critical for understanding tumor biology and developing targeted therapies. In general, the majority of mutations from the primary tumors are retained in their paired metastases, with further enrichment of common somatic SNVs within the protein-altering mutation subset (**Table 3**). For example, 78.6% of somatic SNVs found in P042_PRI were also reported in P042_LIV (77 out of in total 98 somatic SNVs found in P042_PRI) while for protein-altering mutations, the number increases to 88.9% (33 out of in total 36 protein-altering mutation found in P042_PRI), suggesting positive selection pressure on functional alterations. When comparing xenografts to their primary tumors, PDX models (P042_PRI_PDX, P042_LIV_PDX and P047_LIV_PDX) retained the majority of the mutations and also proportionally more of the functional mutations (on average 66.7% and 86.1%, respectively). Both paired primary/metastatic tumors as well as matched patient and xenograft samples have high concordance for somatic SNVs and an even higher concordance for functional alterations.

**Table 3:** Pairwise comparison matrix for common somatic SNVs across all samples.

Patient		P042				P047			P059	
Sample	LIV	LIV_PDX	PRI	PRI_PDX	LIV	LIV_PDX	PER	PRI	LIV_PDX	PRI_PDX
P042_LIV	**233/64**	95/42	77/33	81/34	2/2	2/2	2/2	2/2	1/0	3/0
P042_LIV_PDX		**164/58**	74/31	95/36	2/2	17/4	2/2	2/2	3/0	14/1
P042_PRI			**98/36**	71/31	2/2	2/2	2/2	2/2	0/0	0/0
P042_PRI_PDX				**120/42**	2/2	12/2	2/2	2/2	1/0	6/0
P047_LIV					**91/44**	68/31	60/29	65/31	1/0	0/0
P047_LIV_PDX						**148/52**	67/32	74/31	0/0	7/1
P047_PER							**80/35**	66/29	0/0	0/0
P047_PRI								**93/35**	0/0	0/0
P059_LIV_PDX									**287/87**	158/58
P059_PRI_PDX										**312/99**

In the table, the first number is the total number of common somatic SNVs while the second is the number of common protein-altering ones. The original numbers of somatic mutations and protein-altering ones for each sample are boxed (diagonal).

We extended the comparison to samples from different patients and measured sample similarity using the fraction of common functional SNVs from any two samples (Table 3 and S1 Table). While no common mutations were observed in samples from all three patients, P042 and P047 share two common mutations (**Table 3**). Both mutations are known to be functional: TP53 (R282W) and KRAS (G12V). These findings are not surprising since tumor suppressors such as TP53, SMAD4 and CDKN2A (P16) and oncogenes such as KRAS are frequently mutated in PDAC [4–6]. Interestingly, several mutations were identified in common across distinct PDX models, but were not detectable in the originating patient material. For example (**Table 3**), P047_LIV_PDX has 15 more common mutations with P042_LIV_PDX (17 in total) than with P042_LIV (2 in total) and 10 more common mutations with P042_PRI_PDX (12 in total) than with P042_PRI (2 in total). Though a few of these xenograft exclusive calls might be real, most are likely to be false positives since some mouse reads can map to regions such as ultraconserved elements in the human genome [24, 25] that are almost 100% identical to their mouse orthologous regions and thus could not be fully detected and removed completely before mutation calling.

**Fig 2:**
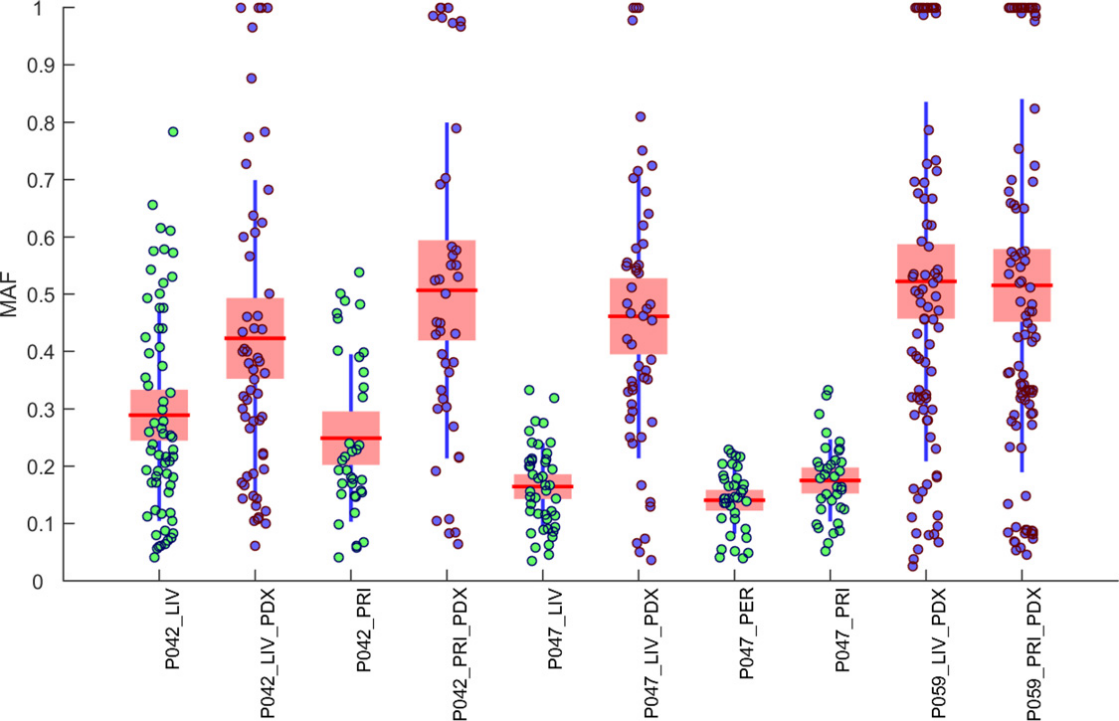
Distribution of MAFs of protein-altering mutations identified in cancer samples. Each boxplot shows the MAFs (from 0 to 1) of somatic SNVs found in an individual sample along with the mean (the red horizontal line), SEM (standard error of the mean, shown a blue box) and SD (standard deviation, shown as a blue vertical line). PDX samples (MAFs in blue dots) in general show higher mean MAF values (close to 0.5) than those from tumor samples (MAFs in green dots), which indicates that the PDX samples were of much higher tumor purity since reads from mouse stromal cells were effectively removed from the original sequencing data.

Besides the number of somatic SNVs, we also compared the distribution of mutant allele frequency (MAF) across all the cancer samples (**Fig 2**). The wide range of MAFs revealed considerable genetic heterogeneity in the samples. While xenograpfts normally have a median MAF close to 0.5, tumor samples present much lower median MAFs (0.15–0.3), most likely due to different level of stromal contamination. For xenografts, a higher median MAF means most sequencing reads from mouse stroma were effectively excluded prior to alignment, so they appear much purer (without reads from mouse stromal cells) than tumor samples (with reads from human stromal cells). This can also explain why in general there are more detectable mutations in PDX samples than in tumor samples (**Table 3**).

To further pinpoint key mutations that might underlie tumor initiation, progression and metastasis, we focused on the predicted functional mutations affecting known cancer drivers and tumor suppressors. **Table 4**lists point mutations affecting TP53, KRAS and SMAD4 while the complete list of protein-alternating mutations can be found in **S1 Table**. As previously described, the R282W mutation in TP53 and the G12V mutation in KRAS are the only two common mutations found in samples from both P042 and P047, while two other TP53 mutations were found in samples from P059.

**Table 4:** Somatic SNVs affecting key cancer genes.

Sample	Gene	PRI	LIV	PRI_PDX	LIV_PDX	AA_change
P042	TP53	0.50	0.50	1.00	1.00	**R282W**
	KRAS	0.40	0.61	0.79	0.77	**G12V**
	SMAD4	0.24	0.42	0.97	1.00	**W524C**
P047	TP53	0.14	0.18	0.17^$^	1.0	**R282W**
	KRAS	0.23	0.21	0.14^$^	0.98	**G12V**
P059	TP53	n.a.	n.a.	0.48	1.00	**P152R**
	TP53	n.a.	n.a.	0.51	0.00	**C182** *

^$^: from P047_PER

*: stop codon.

Besides well-known oncogenic driver mutations, we also found several additional mutations that might be interesting. In samples from P042, a non-synonymous mutation (M9930I) was called for MUC16 in the liver metastatic sample (P042_LIV) but not in the primary sample (P042_PRI). By further visual analysis of available sequencing reads mapping to the MUC16 locus in P042_PRI, we successfully identified two sequencing reads carrying the mutant allele out of a total of 32 reads covering the mutant position, which explained why this SNV was not called in P042_PRI (due to the very low incidences). When checking PDX samples from the same patient, the mutation was also called only in P042_LIV_PDX but not in P042_PRI_PDX. Similarly, by looking into raw sequencing reads from P042_PRI_PDX, we also located two sequencing reads carrying the mutant allele out of 20 reads covering the mutant position. MUC16, also known as CA125 (cancer antigen125), is a mucin, a family of high molecular weight, heavily glycosylated proteins that are known to play important roles in pancreatic cancer pathogenesis [26, 27]. MUC16 is significantly upregulated in pancreatic cancer and co-overexpressed with MSLN (Mesothelin) at the invading edge [28, 29]. It has also been implicated in cell motility and invasion via matrix metallopeptidase 7 (MMP7), and may stimulate cancer metastasis [30]. On the DNA level, MUC16 is frequently mutated in many cancer types, though more likely due to its very large gene size [31]. The mutation M9930I is located in the amino-terminal domain and its functional impact needs to be further studied to see if it plays a role in facilitating pancreatic cancer progression and metastasis. Besides MUC16, there is a deleterious mutation (P1598S, COSMIC Mutation Id[32]: COSM922860) affecting another mucin family gene, MUC2, with elevated MAFs in both metastatic samples (P042_LIV and P042_LIV_PDX) compared to the primary samples (P042_PRI and P042_PRI_PDX). MUC2 has also been implicated in cancer metastasis previously through a tumor-associated macrophage (TAM)-dependent mechanism [33, 34]. Besides P1598S, we also pinpointed two additional protein-altering mutations affecting MUC2 in two metastatic samples from P047 (T1683M in P047_LIV and T1722I in P047_PER, **S1 Table**). While the functional impact of these mutations in mucin proteins remains to be further validated experimentally, the present data suggest that mucin may play a role in helping establish the metastatic founder clone.

In addition to mutations shared between the primary and metastatic tumors, there were predicted functional mutations present in only one or the other tumor. We listed point mutations that appeared to arise *de novo* in the metastatic lesions in **S1 Table**. One interesting gene, ODZ1 is only found to be mutated in metastatic samples (P042_LIV and P042_LIV_PDX). It encodes Tenascin, an oligomericglycoprotein of the extracellular matrix that is a putative tumor suppressor involved in morphogenetic movement, tissue patterning, repair, and tumor invasion, and was reported frequently deleted in malignant gastroenteropancreatic endocrine carcinomas [35, 36]. The mutation N570K, predicted as “Deleterious” by the Sift tool [23], is located in a EGF–like domain and thus might play a role in the development of metastasis by disrupting the gene’s tumor suppressor function. It is possible that, after the physical translocation of cancer cells from primary tumor to distant organ (liver in this case), a subsequent hit on ODZ1 (or other rate limiting events[37]) might have been selected in a subpopulation of tumor cells leading to the formation of a dominant clone at the metastatic site.

**Fig 3:**
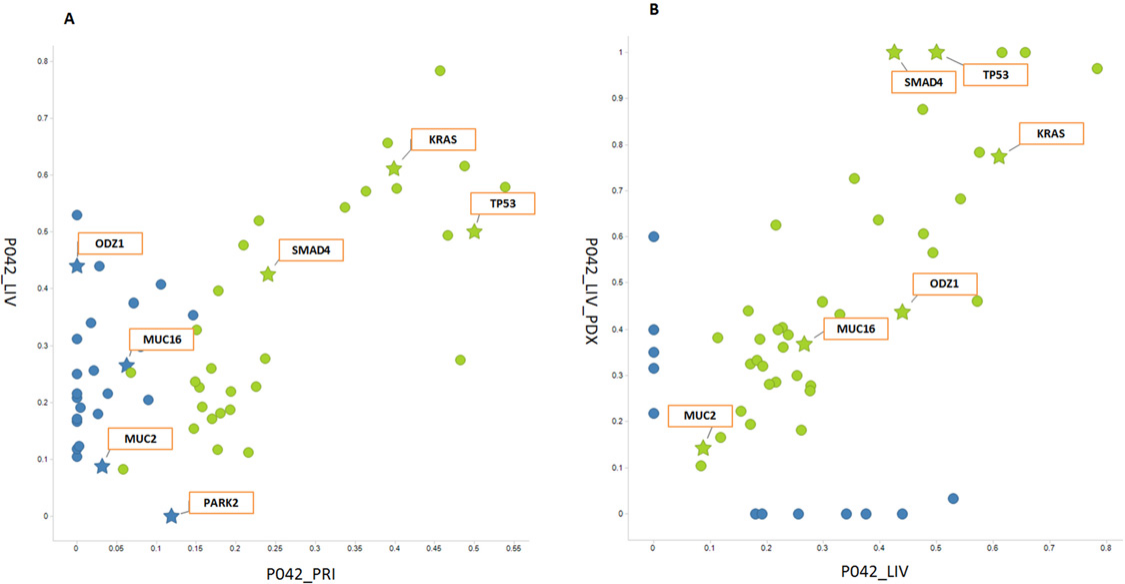
Scatter plots of non-synonymous and non-sense mutations for (A) P042_PRI vs. P042_LIV and (B) P042_LIV vs. P042_LIV_PDX. The plots are based on the mutant allele frequencies (MAFs, from 0 to 1)) of non-synonymous and non-sense mutations from the paired samples. The mutations that are called in both samples are marked by green dots, while ones called in only one sample are colored blue. Gene names are shown for those key mutations discussed in the text.

Comparison of MAF distributions between the primary tumors and their paired liver metastases can help reveal clonal or subclonal mutation patterns underlying PDAC initiation, progression and metastasis. Focusing on protein-altering somatic SNVs, we compared a liver metastasis (P042_LIV) to its xenograft model (P042_LIV_PDX) (**Fig 3A**). The clonal mutations shared between the primary and metastatic tumors (green dots) have a correlated pattern of MAFs, while some subclonal mutations in the primary site have significantly elevated MAFs in the metastatic site, possibly composing the dominant clone in the metastatic tumor. For example, the MUC16:M9903I mutation mentioned previously shows a significant MAF increase from the primary site to the metastatic site in both tumor samples (from 6.3% in P042_PRI to 27% in P042_LIV) and in xenografts (from 10% in P042_PRI_PDX to 37% in P042_LIV_PDX). On the other hand metastasis-specific mutations (the blue dots with MAF (P042_PRI) = 0 in **Fig 3A**) reflect ongoing clonal dynamics in the liver site after metastasis. For primary-specific SNVs, we found only one protein-altering mutation in the gene PARK2 (Parkin RBR E3 Ubiquitin Protein Ligase). It possibly marks a subclone(s) in the primary site that did not migrate to the metastatic site.

Based on the clonal/subclonal mutation information, a parsimonious evolutionary history for P042 can be proposed: first, clonal driver mutations (TP53 and KRAS mutations) appeared early in the primary site (i.e. tumor initiation) followed by subsequent genetic alterations (concurrently or separately) that formed a number of subclones (tumor progression); then a single cell from a minor subclone that acquired an invasive phenotype (marked by mutations such as MUC16:M9930I), migrated to a distant organ (liver in this case) and successfully established a metastasis. We also compared the MAF (**S1 Table**) of the primary tumor samples to their paired metastases from the other two patients (P047 and P059) and found similar patterns of clonal and subclonal mutations. Particularly, in P059, there appeared to be two subclones in the primary site (P059_PRI_PDX) carrying either a missense (P152R, MAF = 48%) or a nonsense (C182*, MAF = 51%) mutation affecting TP53 (**Table 4**). We examined the sequencing reads spanning the two sites and found that the two somatic SNVs are mutually exclusive (data not shown). More interestingly, in the metastatic site (P059_LIV_PDX), it appeared that only the subclone carrying the missense mutation (P152R, MAF = 100%) rather than the non-sense mutation (C182*, MAF = 0%) successfully established the liver metastasis. The apparent hierarchical structure of clones in the primary and metastatic tumors is similar to what had been reported elsewhere [5, 13, 38–40].

We also compared MAF distributions between the tumor samples and their derived xenograft models. **Fig 3B** shows the scatter plot of MAF values for protein-altering mutations in P042_LIV and P042_LIV_PDX. We found that the majority of mutations detected in P042_LIV, including all the key mutations affecting cancer genes such as TP53, SMAD4, KRAS, MUC16 and ODZ1, are retained in the PDX model (green dots in **Fig 3B**). We also noticed a small subset of protein-altering mutations that were either found only in the xenograft or only in the tumor sample. Overall the MAF data demonstrated that the clonal and subclonal mutations from the original tumor are largely retained in xenograft models and this high concordance of somatic alterations indicates that the genomic mutational pattern remains broadly stable in xenografts, consistent with previous reports [17–19, 41]

## Discussion

PDAC patients suffer from rapid disease progression and metastasis, which motivated the search for somatic alterations driving metastasis. In this study, we conducted WES analysis on a collection of rapid autopsy (RA) PDAC primary and matched metastatic tumors and xenografts, and generated a comprehensive portrait of somatic SNVs. Mutations identified from our study in genes such as MUC16, MUC2 and ODZ1 could shed new insights into metastasis-promoting pathways in tumor cells. Although the functional impact of those highlighted somatic SNVs remains to be further tested experimentally and their incidence needs to be determined in a larger cohort, they might serve as a starting point to facilitate our understanding of the molecular mechanisms that underlie metastasis formation in PDAC.

The somatic variants in the PDX models substantially recapitulated their tissues of origin and represent a promising platform to identify and validate potential new pancreatic cancer targets. Based on the somatic SNVs data from this study, we can see that all the key mutations including those on cancer driver genes and tumor suppressors are retained in the xenograft models. Moreover, experimental therapeutic trials in these end-stage derived models should represent a higher bar in which meaningful responses are likely more difficult to achieve but when seen, would generate more confidence to move into the clinic with a new therapy. Overall, these results provide insight into the genetic basis of PDAC metastasis and can help advance the development of targeted therapeutics aimed at controlling progression of pancreatic cancer.

## Materials and Methods

### Patient information

Detailed clinical information can be found in **Table 5.** The rapid autopsy program was established at CNIO for the purpose of harvesting pancreatic cancer patients’ tissue samples within 1–3 hours after death. All samples were obtained with written informed consent. The consent and this study were reviewed and approved by the IRB of Hospital de Madrid, in accordance with the tenets of the Declaration of Helsinki.

**Table 5:** Patient information.

Patient ID	Age (years)	Gender	T/N Stage	Treatment	Met. Site(s)
P042	70	Male	T4N1M1	1st line: Gemcitabine + Abraxane; 2nd line: Cl.Tr. PM 1183-B-001-1; 3rd line: Xeloda	liver
P047	75	Female	T3-4N1M1	Gemcitabine + DLL4 Inhibitor	liver, peritoneum
P059	69	Male	T3-4N1M2	Gemcitabine + Abraxane (4 cycles)	liver

### Sample preparation and whole exome sequencing

Frozen normal, primary tumor and matched metastasis samples were collected and five xenografts were successfully generated from primary tumor or metastases. For P047, the primary tumor xenograft didn’t grow successfully and for P059, not enough materials were found for the primary tumor and liver metastasis. Exome capture was performed using the Illumina TrueSeq exome enrichment kit after DNA sample preparation. The kit targeted for more than 200,000 exons spanning over 62 Mb of human genome (http://images.illumina.com/products/truseq_exome_enrichment_kit.ilmn). Captured DNA from the samples was fragmented and sequenced using HiSeq 2000 sequencer to generate paired end, 100-bp reads. On average, approximately 35–40 million reads were generated and assessed by their quality scores. Low quality reads/bases (Phred score < 20) were removed or trimmed before mapping. For PDX samples mouse reads from the mixture of both human and mouse tissue were removed prior to mapping and mutation analysis using a dictionary based method [42]. This approach identified between 2.4% and17.9% mouse contaminations in the five sequenced xenograft samples. After QC and cleaning of the raw reads, clipped reads were mapped to the hg19 human reference genome using the Burrows-Wheeler Alignment tool (BWA) [43] with default parameters. The mapping quality of the resulting BAM files was inspected, and those with zero quality were filtered out to reduce the false positive mapped reads. PCR duplicates were identified and removed using Samtools [44]. For generating somatic SNV calls, we used Strelka [45] to compare tumor and matched normal samples with a standard Q-score (a measure of the confidence of the somatic calls [45]) to be equal or greater than 15**.** Then the somatic SNVs were annotated based on the ENSEMBL[46] and their functional impact was predicted by the SIFT tool[23]. The raw data from this study has been made available through the European Nucleotide Archive (ENA), with accession number: PRJEB9296. (http://www.ebi.ac.uk/ena/data/view/PRJEB9296).

## Supporting Information

S1 Table (XLSX)
